# Tool for Semiautomatic Labeling of Moving Objects in Video Sequences: TSLAB

**DOI:** 10.3390/s150715159

**Published:** 2015-06-29

**Authors:** Carlos Cuevas, Eva María Yáñez, Narciso García

**Affiliations:** Grupo de Tratamiento de Imágenes, Universidad Politécnica de Madrid (UPM), E-28040 Madrid, Spain; E-Mails: eyg@gti.ssr.upm.es (E.M.Y.); narciso@gti.ssr.upm.es (N.G.)

**Keywords:** ground-truth, labeling, moving object detection, tracking, active contours, shadows, occlusions, tool

## Abstract

An advanced and user-friendly tool for fast labeling of moving objects captured with surveillance sensors is proposed, which is available to the public. This tool allows the creation of three kinds of labels: moving objects, shadows and occlusions. These labels are created at both the pixel level and object level, which makes them suitable to assess the quality of both moving object detection strategies and tracking algorithms. The labeling can be performed easily and quickly thanks to a very friendly graphical user interface that allows one to automatize many common operations. This interface also includes some semiautomatic advanced tools that simplify the labeling tasks and drastically reduce the time required to obtain high-quality results.

## 1. Introduction

The number of computer vision applications has exponentially grown during the last few years [1,2], mainly due to the huge amount of consumer electronic devices with optical sensors that claim them, such as smart-phones, tablets, video game consoles or intelligent vehicles [3].

Parallel to the appearance of these applications, many public datasets have been proposed to assess the quality of their results [4,5], which could be classified according to the type of application for which they have been created: semantic classification [6], text recognition [7], moving object detection [8], human motion [9], tracking [10], facial recognition [11], *etc.*

Among these datasets, those focused on testing the results of the strategies for moving object detection and tracking are very relevant, since these strategies are considered critical steps in many high-level tasks (*i.e.*, people counting, object classification, augmented reality, gesture recognition, *etc.*) [12–14]. However, to obtain this kind of dataset, the sequences must be labeled at both the pixel level and object level, which is extremely costly.

In spite of the interest of many researchers in the development of new and complete databases to assess the quality of their moving object detection and tracking algorithms, to the best of our knowledge, there are no public tools specifically designed to perform the labeling of moving objects in video sequences. Consequently, most of the existing datasets are incomplete: they only provide ground-truth data for some frames [15,16] or for some specific regions of interest in the images [8].

The only existing public tool that could be used for this purpose is LabelMe Video [17], which is an online tool that allows one to annotate forms and names of any kind of object in an image, as well as events. However, this tool is not focused on labeling moving objects at the pixel level and along all of the images in a video sequence.

In this paper, we propose a new and user-friendly labeling tool named TSLAB (Tool for Semiautomatic Labeling), which is available to the public on a website [18]. This tool allows one to efficiently create ground-truth data of video sequences at the pixel level and at the object level. Therefore, it is perfectly suitable for researchers that require creating ground-truth to evaluate their object detection and/or tracking algorithms. The semiautomatic denomination in the name of the proposed tool refers to the fact that the labeling can be done using different automatic mechanisms that must be supervised by the user to ensure the required quality.

TSLAB provides some useful mechanisms to accelerate the labeling along consecutive images of a video sequence (e.g., the automation of common operations or the automatic labeling of a moving object remaining static temporally). Moreover, it includes some advanced tools (e.g., an active contours-based method and some motion detection algorithms), which significantly simplify the labeling and, in some cases, even allow a fully-automatic segmentation. Additionally, with the aim of being able to provide ground-truth data suitable for strategies that try to detect and/or delete shadows cast by moving objects or tracking methods that require knowing not only the visible regions of moving objects, but also their occluded ones, TSLAB allows the labeling of three kinds of data: moving objects, shadows cast by moving objects and occluded regions of moving objects.

The paper is organized as follows. Section 2 describes the data structure of TSLAB. In Section 3, its interface and main functions are presented. Sections 4–6 describe the semiautomatic tools included in TSLAB: the motion detection tool in Section 4, the intelligent pencil tool in Section 5 and the active contours tool in Section 6. The employed software, the format of the data used and the output formats are detailed in Section 7. Finally, Sections 8 and 9 summarize, respectively, the results and the main conclusions.

## 2. Data Structure

**Figure 1:**
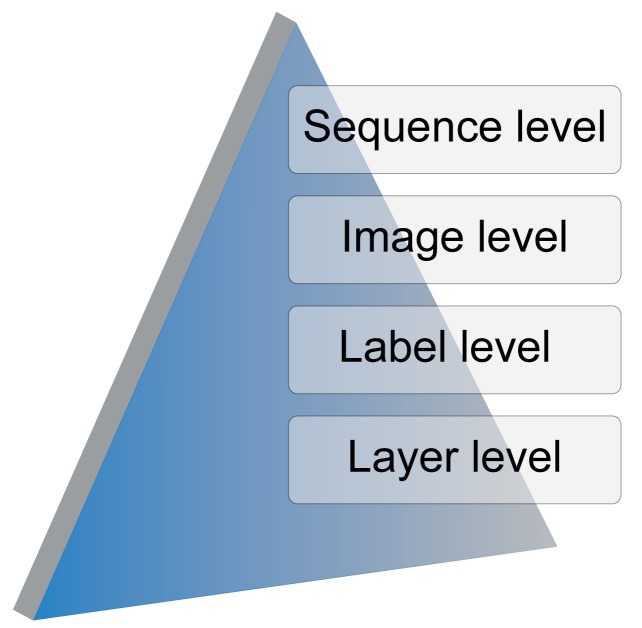
Hierarchy levels in the architecture of the Tool for Semiautomatic Labeling (TSLAB).

TSLAB has been designed hierarchically according to the levels illustrated in Figure 1:

- Sequence level: This allows one to select the video sequence to label and, if it exists, a set of stored ground-truth data to use as the starting point. If a previously-created ground-truth is not available, a directory for the new labeled data is created.
- Image level: This allows one to select the image to label in a video sequence.
- Label level: For a selected image, this allows one to select, create or modify an existing label.
- Layer level: This allows one to navigate across the different layers (visible area, shadow or occluded area) of a selected label.

### 2.1. Label Level: Pixel-Based and Object-Based Labeling

The ground-truth data required to evaluate moving object detection methods are different from those required to perform the quality of tracking algorithms. On the one hand, motion detection techniques try to identify what pixels or regions of an image belong to the moving objects [19], but they do not establish any relation among such pixels or regions. Therefore, to assess the quality of their results, they require pixel-level binary masks indicating what pixels are part of the moving objects [20]. On the other hand, tracking techniques try to determine the number of moving objects at each image and their trajectories along the sequences. Consequently, these methods are commonly evaluated with datasets consisting of bounding boxes around the moving objects [21].

TSLAB provides a specific label to each moving object at the pixel level. Therefore, its output data can be used by both detection and tracking methods.

### 2.2. Layer Level: Moving Objects, Shadows and Occlusions

The proposed labeling tool distinguishes between three types of ground-truth layers for the labeled moving objects:

- Visible moving objects (VMO): This layer is assigned to all of the visible pixels of the moving objects.
- Shadows from moving objects (SMO): This layer is assigned to the pixels belonging to shadows cast by the moving objects.
- Occluded moving objects (OMO): This layer is assigned to non-visible pixels of the moving objects (occluded regions).

**Figure 2:**
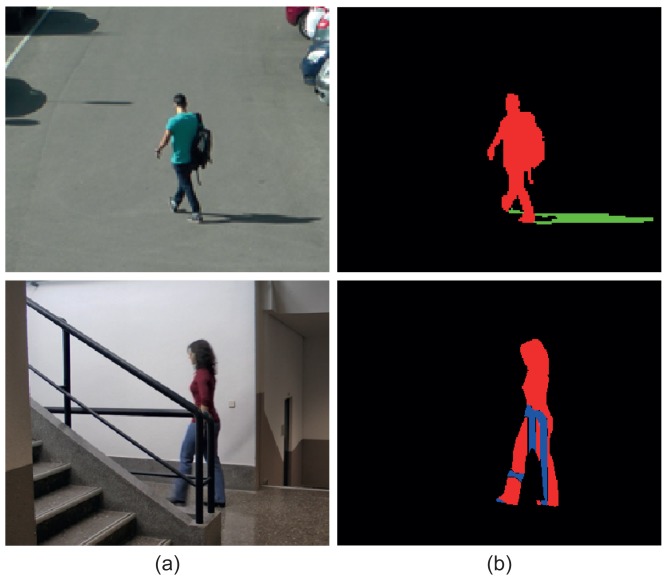
(**a**) Two original images; (**b**) masks of layers. Color notation: visible moving objects (VMO) (red), shadows from moving objects (SMO) (green) and occluded moving objects (OMO) (blue).

Figure 2 shows two original images and their corresponding ground-truth masks, which contain the above-described layers. It can be observed that the top mask presents a VMO layer and a SMO layer, while the bottom mask contains a VMO layer and and OMO layer.

All of the pixels of a moving object will be always distributed in these three layers. However, more than one layer can be assigned to one pixel. For example, one pixel can be classified as VMO if it is part of a visible region of a moving object, but it can also be classified as OMO if it is part of an occluded region of another moving object.

As described in Section 7.2, for each labeled image, TSLAB creates a 24-bpp RGB image. The data corresponding to the VMO, SMO and OMO layers are contained, respectively, in the R, G and B channels of these images. The zero value in each channel is reserved to indicate that there is no layer, while the remaining 255 values are used to indicate the image-level identifiers of the labeled moving objects. These identifiers are assigned to the objects in order of labeling. For instance, a pixel with the value (2, 1, 0) indicates that such a pixel belongs to the VMO layer of the second label and to the SMO layer of the first label.

**Figure 3:**
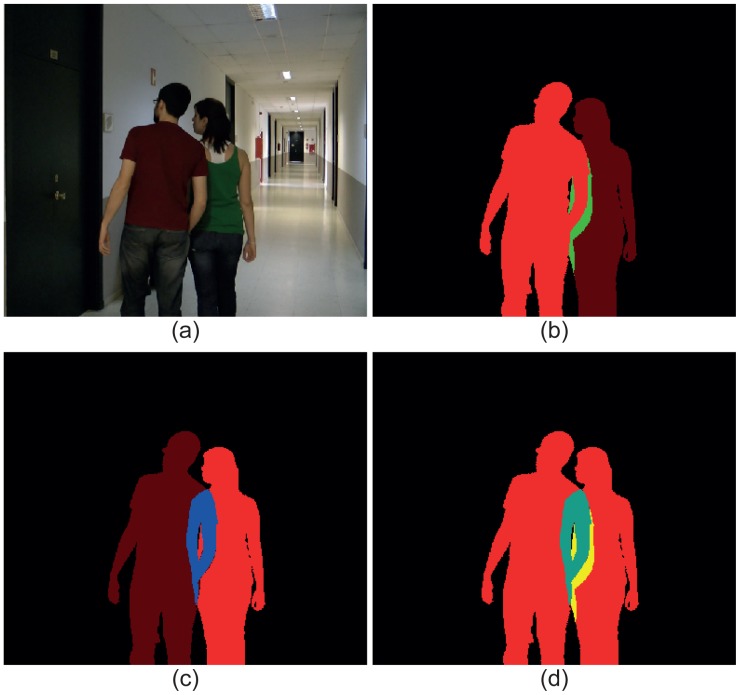
Layers obtained from an image with two moving objects partially occluded. (**a**) Original image; (**b**) layers of the left object (VMO in red and SMO in green); (**c**) layers of the right object (VMO in red and OMO in blue); (**d**) layers of both objects (VMO in red, SMO in yellow and OMO in cyan).

Figure 3 illustrates an image with two moving objects (Figure 3a) and three ground-truth masks containing the three possible types of layers. In Figure 3b, the data corresponding to the moving object in the left have been highlighted: red for the VMO layer and green for the SMO layer. As a visual reference, the silhouette of the other moving object has been also included in this mask. Analogously, the layers corresponding to the moving object in the right of the image have been highlighted in Figure 3c: red for the VMO layer and blue for the OMO layer. Again, the silhouette of the other moving object has been included as a visual reference. Finally, Figure 3d depicts the total mask, where the red pixels indicate that they belong to VMO layers, the cyan pixels indicate that they are part of both a VMO layer and a OMO layer (the sum of red and blue is cyan) and the yellow pixels indicate that they contain data from a VMO layer and a SMO layer (the sum of red and green is yellow).

## 3. Graphical User Interface and Basic Functions

The graphical user interface of TSLAB has been designed to be user friendly and very intuitive. It has two main windows: a navigation window and a labeling window.

**Figure 4:**
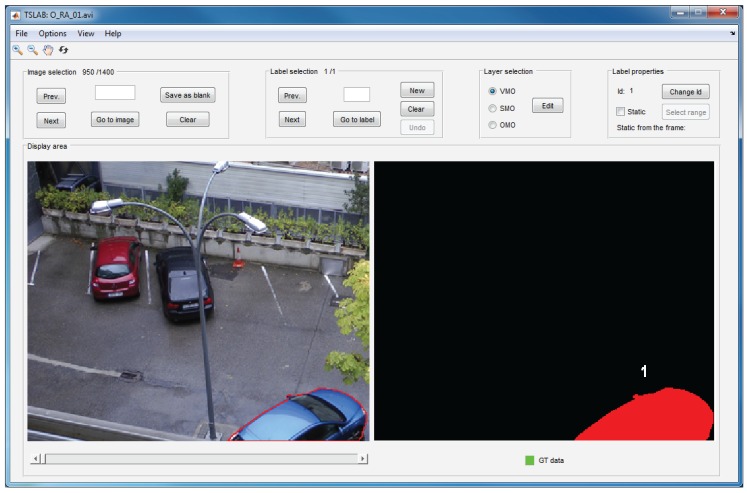
Navigation window.

The navigation window, which is illustrated in Figure 4, is composed of a main menu, a toolbar and five panels: image selection, label selection, layer selection, label properties and display area. The main menu includes functions that allow one to load sequences, reset or exit the application, saving the ground-truth data in the formats specified in Section 7.3, or choosing the content to visualize in the display area. The five aforementioned panels allow one to navigate across the loaded sequence (image selection panel), navigate across the labels into an image (label selection panel), choose the layer to edit (layer selection panel), change the identifier of a label or set a label as a static object (label properties panel) and visualize the original images and the created labels (display area panel). Finally, the toolbar (located below the main menu) can be used to enlarge or reduce the visualized images, to scroll the viewing area and to return the initial view. The details concerning the use of all of these menus and panels appear in the user manual, which can be downloaded from the same website that TSLAB [18].

**Figure 5:**
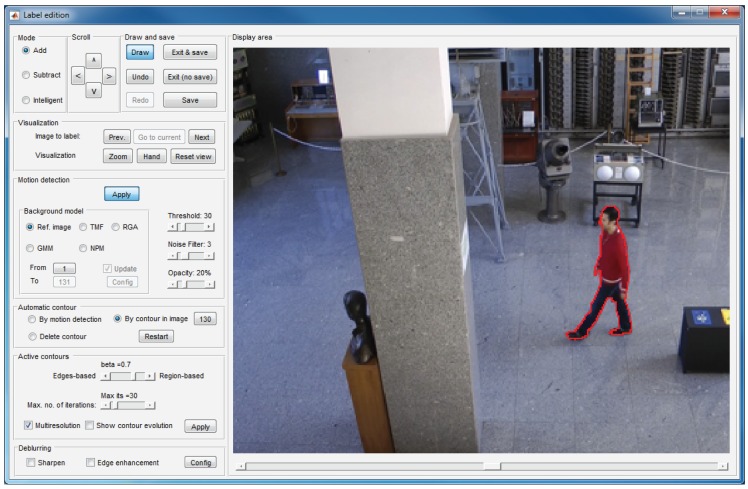
Labeling window.

The labeling window, which is illustrated in Figure 5, is formed by nine panels that allow one to create and edit layers on the current image.

Some of these panels allow basic functions: draw contours on the current image (display area panel), select the drawing mode (mode panel), scroll a contour drawn on the image (scroll panel), undo or redo previous operations, save the work or exit the window (draw and save panel) or visualize other images to facilitate deducing whether an image area belongs to a moving object or to the background (visualization panel). Among these functions, noteworthy is the intelligent mode in the mode panel, which is described in Section 5.

**Figure 6:**
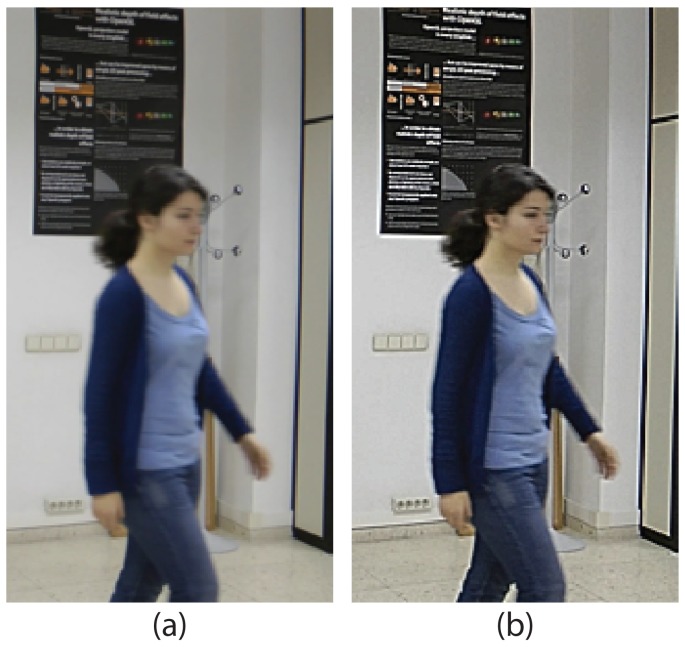
Example of use of the deblurring tools. (**a**) Original image; (**b**) deblurred image.

The remaining panels allow one to apply different tools focused on reducing the labeling effort:

- Motion detection panel: This allows one to activate the motion detection tool (detailed in Section 4).
- Automatic contour panel: By using the options in this panel, it is possible to get an initial contour that can be refined or displaced to arrive at the target contour. If “By motion detection” is selected, the initial contour is that resulting from the motion detection tool (Section 4). If “By contour in image” is selected, the initial contour is the contour created for the image indicated in the panel. This second option is very helpful in the labeling of solid-rigid moving objects, since these objects maintain the form of their contours along the sequence.
- Active contours panel: This panel can be used to activate and configure the active contours tool (detailed in Section 6).
- Deblurring: The options in this panel allows one to enhance the edges of the objects, which facilitates the labeling task when the image is blurred. Figure 6 shows an example in which the use of the deblurring options is very useful.

Similarly to the case of the navigation window, the details regarding the use of all of the functions included in the labeling window appear in the user manual that can be downloaded along with TSLAB [18].

## 4. Motion Detection Tool

This tool allows one to estimate a background model that can be compared to the current image to identify the moving objects in such an image. In many cases, the moving regions resulting from these comparisons are very good approximations of the target labels, and only some small changes must be manually done to finish the labeling work.

TSLAB helps to detect moving objects in five ways:

- Using another image as the background: This is the fastest option. However, it requires a careful choice of the background image (it must not contain illumination changes, and the object to segment should not appear in it). Additionally, this option is only useful in sequences with a very static background.
- Applying temporal median filtering (TMF) [22]: This option allows one to construct a reference background image as the median of a set of consecutive images selected by the user. The result of comparing the current image and this background model is robust to typical image noise due to the camera sensor. In addition, if the range of images used to construct the model is large enough, the results obtained can be satisfactory even in the presence of moving objects in such a range of images. However, the computational cost of this method increases, as does the number of reference images.
- Applying the running Gaussian average (RGA) method [23]: This method tries to fit a Gaussian probability density function on the most recent samples of each image pixel. Over the last few decades, it has been widely used by many authors as a starting point to detect moving objects [24,25], since it is able to provide successful results in short sequences with quasi-stationary backgrounds, and its computational and memory requirements are low. However, it is not efficient in some critical situations, such as illumination changes or dynamic backgrounds.
- Using Gaussian mixture models (GMM) [26]: A mixture of several Gaussians is used to obtain an adaptive model of each one of the image pixels. Therefore, it is possible to model more than one state for each pixel, thus classifying correctly the background areas containing elements with cyclic movements. Because of this property, GMM-based methods have probably been the most used over recent years to detect moving objects [27–29]. However, these methods depend on several parameters [30], which decrease their usability. Additionally, they are not flexible enough to model background complex density functions. Therefore, they fail in sequences with very dynamic backgrounds [31].
- Using non-parametric modeling (NPM) [12]: In this case, in contrast to the previous methods, that the values of the pixels belong to a particular distribution is not considered, and a probabilistic representation of the observations is constructed using a recent sample of values for each pixel. This strategy improves the results provided by RGA and GMM, and additionally, its usability is very high (it depends on few parameters). However, its main drawback is that it involves very high memory and computational requirements [32].

On the one hand, if one of the first two options is used, the obtained reference image, *B*, should be compared to the current one, *I*, to construct a binary mask of differences:

(1) $$D_{B,I}(x,y)=\{\begin{array}{cc} 1 & \text{if}∥B(x,y)-I(x,y)∥>Th\\ 0 & \text{if}∥B(x,y)-I(x,y)∥\leq Th \end{array}$$

where (*x*, *y*) are the pixel coordinates, ‖ · ‖ is the euclidean distance and *Th* is a threshold that must be selected by the user in the “Motion detection” panel. If the threshold value is too large, many areas of the moving object will be not detected. However, if its value is too small, not only the moving object will be detected, but also the shadows cast by it.

On the other hand, the last three options (RGA, GMM and NPM) provide, directly, a binary mask of moving objects. Therefore, they do not require the selection of thresholds, which simplifies the use of the motion detection tool.

Finally, to delete small regions and to make the detected objects more compact, an opening morphology operation followed by a closing morphology operation are applied on the binary mask. The structuring element used to perform these operations is circular, and its size can be determined by the user. If the moving object to segment is small, this value must be small also, but for large moving objects, it can be higher.

The choice of using one method or another will depend on the characteristics of the sequence and on the user requirements. For example, in simple sequences with a very static background, the most appropriate would be to use another image as the foreground (if the camera noise is low) or the TMF (if the camera noise is significant), since these methods are fast and they only require setting one parameter (the threshold *Th*). However, if the sequence to label contains a complex background (with non-static elements or illumination changes), the use of RGA, GMM and NPM would be adequate. On the one hand, RGA is the best option if the background changes are slow, since it depends on few parameters and is fast. On the other hand, GMM and NPM must be selected if the background is multimodal. The first one is faster, but it depends on several parameters that must be set according to the sequence characteristics. The second one is much slower, but it is the least dependent on parameters. Moreover, it must be noted that these three last options will not provide successful results at the beginning of the sequences, since they require a large set of training images to work correctly. Therefore, its use is not recommended in the first images of the sequences.

All of these methods have been configured with those default values that usually provide the best quality detection in a great amount of test sequences. However, they can be modified by the user to try to improve such detections.

In addition, the RGA, GMM and NPM options have been equipped with the possibility of using some improvements that are focused on reducing the influence of shadows in the results and to deal with sequences recorded with non-completely static cameras.

**Figure 7:**
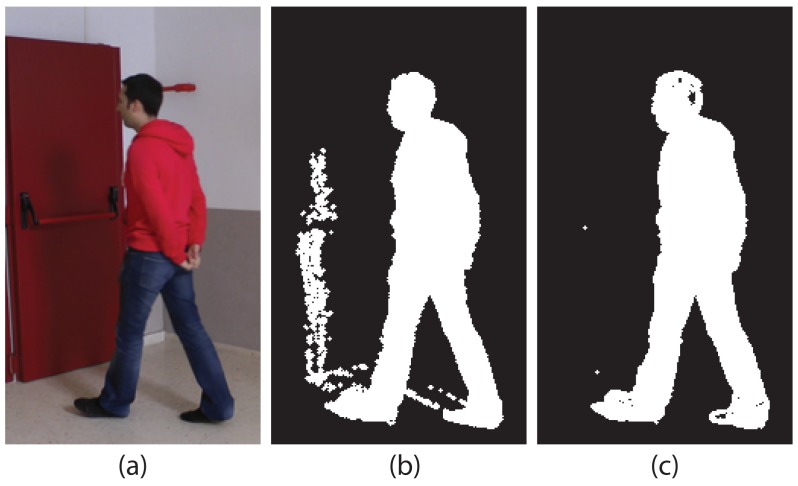
(**a**) Original image; (**b**) detection using RGB color; (**c**) detection using the alternative appearance components (*RnGn*| ∇ *s*|).

On the one hand, TSLAB does not only allow the user to configure these methods to use the typical RGB color components, but it also allows one to select the set of appearance components proposed in [31], which has proven to be very robust to shadows cast by moving objects and to soft illumination changes. This set of components is composed by the red and green chromaticities (*Rn* = *R*/(*R*+*G*+*B*) and *Gn* = *G*/(*R* + *G* + *B*)) and by the modulus of the gradient of the brightness (| ∇ *s*|, where *s* = R+G+B). Figure 7 shows some results using the typical RGB color and the alternative appearance components. It can be seen that, using this alternative, the shadows cast by the moving object have been drastically reduced, which will facilitate the labeling of the moving object.

On the other hand, the NPM method also includes the option of applying a spatio-temporal modeling that is able to correctly model small displacements of the background [12], thus reducing most false detections in sequences with jitter or slow camera panning.

## 5. Intelligent Pencil Tool

Usually, the motion detection tool is not enough to label the moving objects, since it is very difficult to detect the moving objects correctly while avoiding the shadows that they cast. However, combining it with the intelligent mode, it is possible to achieve accurate results very easily.

**Figure 8:**
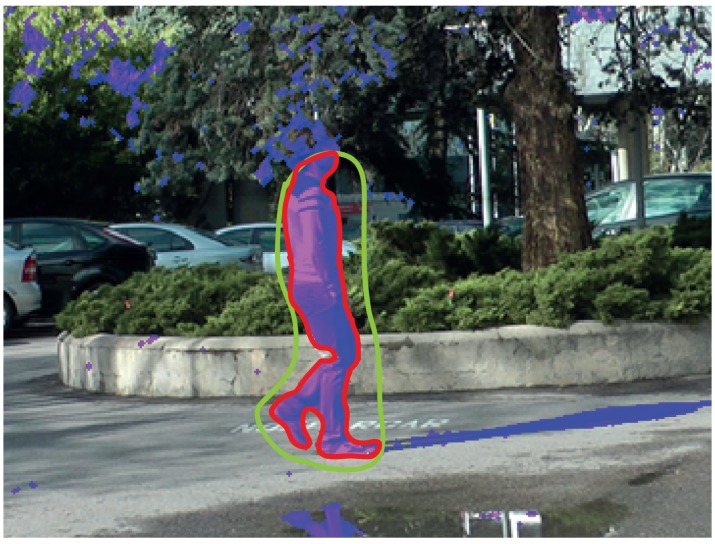
Example of using the intelligent mode combined with the motion detection tool. The difference mask is shown in semitransparent purple. The contour drawn by the user with the intelligent mode is shown in red. The contour resultant from the combination of the motion mask and the contour manually drawn is shown in red.

If the mode “Intelligent” is selected in the labeling window, it is possible to select (by drawing on the display area) what parts of the mask of differences will become part of the label. Figure 8 illustrates an example where the combination of the motion detection tool and the intelligent mode provides a satisfactory labeling. As shown in this example, the mask resulting from the application of the motion detection tool (depicted in purple in the image) contains many unwanted regions (dynamic background areas and shadows). However, the intelligent mode allows one to easily select the area that we want to include as part of the label (area inside the green contour): the resulting region is the intersection between the area obtained by the motion detection strategy and the area inside the contour drawn with the intelligent pencil. After carrying out this selection, it is seen that a very good approximation to the target contour has been obtained (red contour in the image). In this example, to complete the labeling, it is only necessary to manually refine the area around the head of the moving object.

## 6. Active Contours Tool

Active contours are a very powerful tool for detecting contours, especially if we have an initial approximation of the contour that we want to reach. Therefore, to facilitate the labeling task, we have included in TSLAB the active contour-based segmentation strategy proposed in [33].

This strategy, taking as a starting point the algorithms described in different keynote active contours strategies [34–36], proposes to minimize a combination of boundary-based and region-based energies in a local neighborhood along the curves. In this way, it is able to obtain the best overall quality in a large number of scenarios. Moreover, to reduce the computational cost of the segmentation process and to improve its usability (which are the main drawbacks of the strategies that get the best overall results), it applies a very efficient multiresolution analysis. Therefore, this strategy is suited perfectly to TSLAB, since it is not only capable of providing high-quality results in many scenarios, but also depends on few parameters and is very fast.

The parameters in the “Active contours” panel have been set with those default values that usually offer the best results. However, they can be modified by the user to try to improve such results. The *β* parameter can be set with values between zero and one and defines the contribution of each kind of energy used by the segmentation method. If *β* = 0, the contribution of the boundary-based energy is maximum, whereas if *β* = 1, the region-based energy contribution is maximum. Regarding the maximum number of iterations, if the starting contour is far from the target contour, this number must be high. However, it must be noted that the speed of the algorithm decreases as the number of iterations increases. If the multiresolution is enabled, the computational cost of the algorithm will decrease considerably. However, disabling it, the quality of the results could be improved in some situations (e.g., in scenarios with complex background, composed by many small regions).

**Figure 9:**
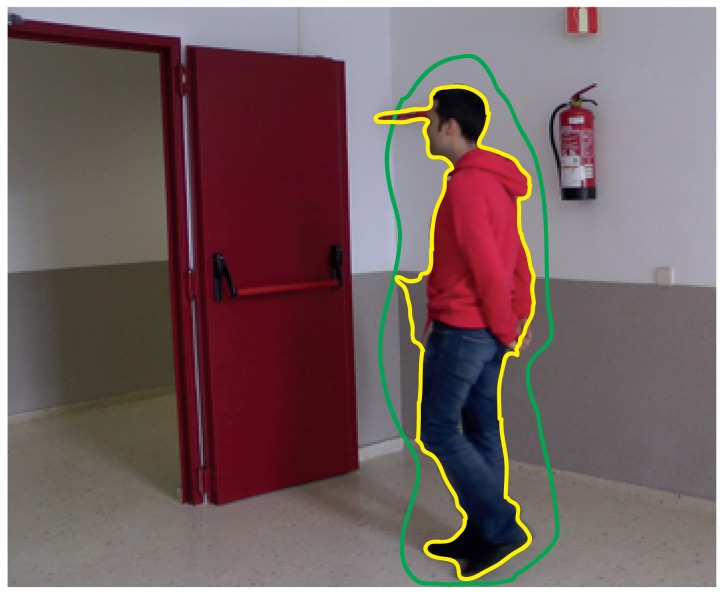
Example of using the active contours mode. The green line illustrates the initial contour. The yellow line shows the final contour.

Figure 9 shows the result obtained by using the active contour-based method integrated in TSLAB. The green line corresponds to the initial contour (manually drawn by a user). The yellow line corresponds to the contour finally obtained. Although the initial contour is very imprecise and also is away from the target contour, it can be observed that the achieved contour is very precise and only requires small adjustments in some areas.

## 7. Software and Data Format

TSLAB has been performed using MATLAB [37], which is a widespread software in the scientific community that uses a high-level interpreted language and offers a great amount of computer vision functions and tools.

When the labeling of a new video sequence starts, a file of metadata is created in the ground-truth folder. The content of this file is described in detail in Section 7.1.

In addition to this metadata file, for each labeled image, a ground-truth image is also created in the ground-truth folder. These images, which are described in detail in Section 7.2, will contain the details of the labels in each image.

From these data (metadata and ground-truth images) TSLAB offers the possibility to generate many output data formats, which are detailed in Section 7.3.

### 7.1. Metadata File

The metadata file contains two matrices with different types of information:

- seqMatrix: This contains information on the labeling performed for each of the images in the sequence (each row corresponds to one image). The data collected for each image, which is distributed in columns, are shown in Table 1.
- objMatrix: This is a three-dimensional vector that contains information about the moving objects labeled in the video sequence. Its first dimension specifies the number of images; the second dimension specifies the number of moving objects (from one to the number of labels in the image); and the third dimension contains different data related to such a moving object in such an image (these data are detailed in Table 2).

With the exception of *Id*, *AltId* and *N-Sta*, which only can be known using the second of these matrices, the rest of the data can be deduced from the ground-truth images described in the following section. However, it is very helpful to have these data accessible without requiring loading and analyzing the ground-truth images.

### 7.2. Ground-Truth Images

For each labeled image, a 24-bpp RGB ground-truth image is created, whose R, G and B channels are used to store, respectively, the VMO, the SMO and the OMO layers. Non-labeled pixels will have the value zero in the three channels. On the contrary, labeled pixels will have, in the corresponding channel, the image-level identifier assigned to the labeled object (values from one to *N* that have been assigned to the labels in creation order).

### 7.3. Saving Options

**Figure 10:**
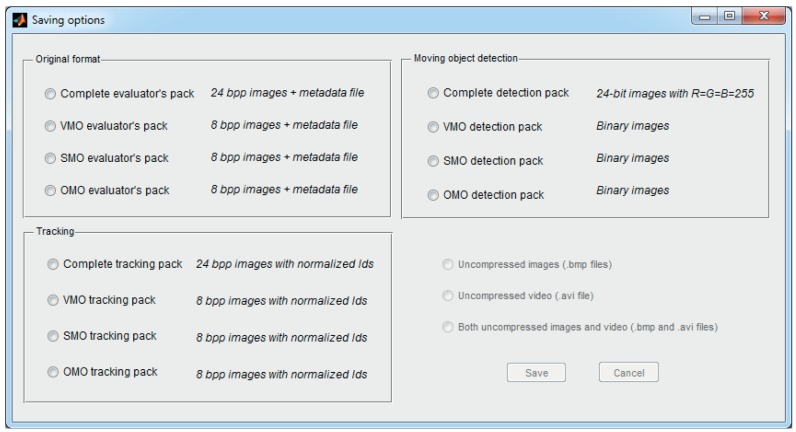
Saving options included in TSLAB.

To adapt the ground-truth data to the needs of the different users, TSLAB offers multiple options to save these data, which can be accessed from the menu in the navigation window. The offered saving options are illustrated in Figure 10, where it can be observed that these are distributed in the following three panels:

- Original format: This options allow one to save the metadata file and the ground-truth images described in Sections 7.1 and 7.2. The values of the pixels in the ground-truth images, instead of the numbers assigned in the labeling process (as stated in Section 7.2), contain the *Id* values of the labels.
- Tracking: The options in this panel discard the metadata file, and similarly to the previous panel, they modify the ground-truth image to contain the *Id* values. Additionally, to facilitate the visualization of these labels, these identifiers are normalized as:
  (2) $$Id_{\text{norm}}=255-\left\lfloor \frac{255}{N}\right\rfloor (Id-1)$$
- Moving object detection: The saving options in this panel discard the metadata file and the object-level data, thus providing pixel-level masks. As mentioned in Section 2, this type of datum is commonly used to assess the quality of moving object detection methods.

Each of these three panels allows saving all of the R, G and B channels of the images (containing, respectively, the data of the VMO, the SMO and the OMO layers) or saving only one of these channels (only the data corresponding to one of the layers).

## 8. Results

To test the effectiveness of TSLAB, we have carried out some quality and efficiency tests with 20 users (12 men and eight women) aged between 21 and 35 years with a mean age of 28 years. These tests have been carried out on a 3.60-GHz CPU with 16 GB RAM, and they consisted of the labeling of two images under the following conditions:

- Test 1: Using only manual tools.
- Test 2: Using as a starting point the contour created for the previous image.
- Test 3: Using the motion detection tool (Section 4) to obtain an initial contour (selecting the option “By motion detection” in the “Automatic contour” panel).
- Test 4: Using the motion detection tool (Section 4) to obtain an initial contour and the intelligent pencil tool (Section 5) to select the areas of interest.
- Test 5: Using as a starting point the contour created for the previous image and applying the active contours tool (Section 6).

**Figure 11:**
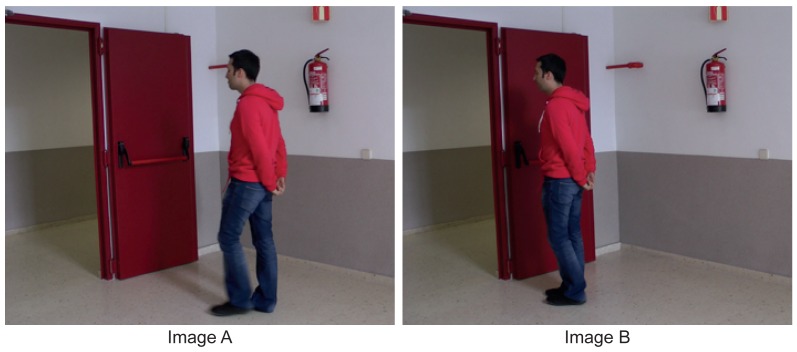
Images used to evaluate the effectiveness of TSLAB.

The tests have been performed on the two images depicted in Figure 11. These images, despite belonging to the same sequence, have very different characteristics. On the one hand, the color of the moving object in Image A is very different from the background on which it is located. Moreover, its displacement relative to the previous image in the sequence is significant. On the other hand, the moving object in Image B has almost stopped, and it is placed on a background region with a color similar to its own.

The evaluation of the quality has focused on comparing the results obtained by the users with labels that have been obtained (very carefully and by using exclusively manual tools) by an expert user of TSLAB. These comparisons have been carried out using the *F* parameter [38], which evaluates jointly the recall (relates the amount of correctly-segmented pixels with the number of segmented pixels in the ground-truth) and the precision (relates the number of correctly-segmented pixels with the amount of segmented pixels).

**Figure 12:**
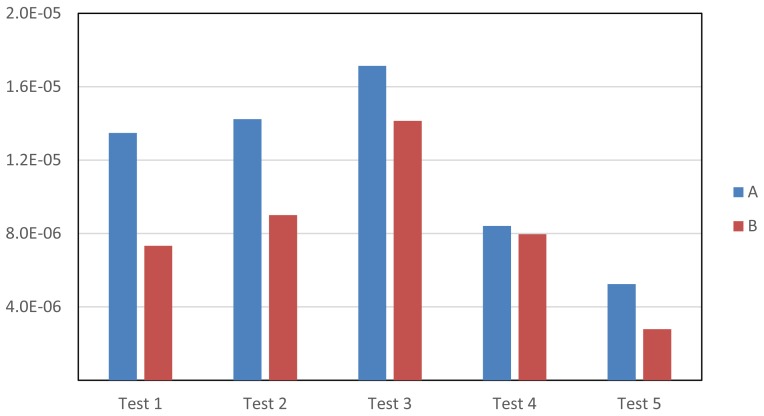
Variance of the *F* values for the performed experiments.

Figure 12 shows the variance of the *F* values obtained for each test and each image. It can be observed that the variance is lower for Image B in all of the experiments. This is because the edges of the moving object in Image A are blurred (due to its movement), and this leads to a wide variety of criteria when labeling. The results in this figure also show that in all of the experiments (the five tests and the two images), the results provided by all of the users are very similar (very low variance values). This demonstrates that the different tools included in TSLAB barely influence the quality of the results. As shown below, the use of one tool or another affects only the time required to segment the objects.

**Figure 13:**
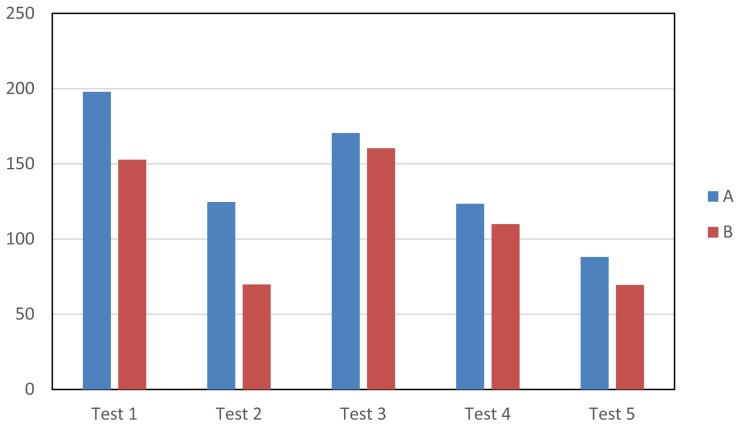
Mean time values (seconds) expended on the performed experiments.

Figure 13 illustrates the mean time values spent on each test and for each image. It can be observed that all of the advanced tools (Tests 2–5) give rise to a reduction of the time required to label (compared to the time spent on Test 1, in which only manual tools are used). In Test 2, where the labeling starts from the contours obtained for previous images, a significant reduction has been obtained for both images. This reduction is higher for Image B, since in this image, the object barely moves. The lowest computational reduction has been obtained in Test 3. This is due to using a motion detection mask, so that it is very difficult to obtain an initial contour with enough accuracy, since the edges of the masks are frequently inaccurate and many reflects cast by the moving objects are usually included as part of the detection. Consequently, users must apply many corrections to obtain the final labeling. However, as is seen in Figure 13, in Test 4, where the motion detection tool is combined with the use of the intelligent pencil tool, an additional reduction of the time required for the labeling has been achieved. This is because the intelligent pencil allows one to easily select the valid areas from the motion detection mask and, simultaneously, to draw accurate contours in those areas where the motion detection fails. Finally, it can also be observed that the active contours tool (Test 5) provides the best results (the lowest computational costs) in both images, since it applies a very fast algorithm that is able to provide a very good approximation to the labeling targets. Therefore, very few changes must be done by the users after using this tool.

Once the tests were completed, users were asked to rate, from one (bad) to five (good), the usefulness of the tools used in all of the tests. The results of this subjective evaluation (means and variances) appear in Table 3. It can be noted that all of the tools were rated with quite acceptable notes and that these notes are inversely proportional to the time required to finish the labeling. Additionally, the users were asked about their favorite tool. Thirteen users chose the active contours tool; four users chose the motion detection tool combined with the intelligent pencil; and three users chose the option of selecting a contour from a previous image as a starting point.

Finally, the users were also asked to rate (from 1–5) some general aspects of TSLAB, and all of the results were very good. The average rates of their answers are the following:

- Usefulness: 4.85
- Usability: 4.6
- Performance: 4.4
- Overall assessment: 4.6

## 9. Conclusions

A new and user-friendly tool, the Tool for Semiautomatic Labeling (TSLAB) of moving objects in video sequences, has been presented; it is available to the public at [18].

This tool is able to provide both pixel-level and object-level ground-truth data. Additionally, it considers three possible types of labels: visible moving objects, shadows cast by moving objects and occluded moving objects. Therefore, it can be used to generate data to assess the quality of a wide variety of algorithms to detect and/or track moving objects.

The proposed tool has a very intuitive graphical interface that allows one to automatize many common labeling operations. Additionally, it includes some high-level analysis options (e.g., background subtraction methods and active contour-based algorithms) that drastically simplify the labeling tasks and help with being consistent in the labeling of the moving objects across the video sequences.

The effectiveness of the labeling tool has been proven by some quality and efficiency tests that have been carried out on many users with heterogeneous profiles. These tests have shown that by using the proposed tool, it is possible to significantly reduce the time required to label the moving objects, while allowing one to obtain accurate labels.

## Figures and Tables

**Table 1:** Fields in seqMatrix.

**No. of Column**	**Code**	**Description**
1	*GT*	If its value is 1, this indicates that the image has been labeled.
2	*N*	This indicates the total number of labels in the image.
3	*seqVMO*	If its value is 1, this indicates that there are VMO labels.
4	*seqSMO*	If its value is 1, this indicates that there are SMO labels.
5	*seqOMO*	If its value is 1, this indicates that there are OMO labels.
6	*Sta*	If its value is 1, this indicates that there moving objects labeled as static.

**Table 2:** Fields in objMatrix.

**No. of Column**	**Code**	**Description**
1	*Id*	Global identifier of the object (in order of appearance in the sequence).
2	*AltId*	Alternative object identifier. Unlike Id, this allows one to sort the moving objects according to the criteria desired by the user.
3	*objVMO*	If its value is 1, this indicates that the object has VMO labels in the image.
4	*objSMO*	If its value is 1, this indicates that the object has SMO labels in the image.
5	*objOMO*	If its value is 1, this indicates that the object has OMO labels in the image.
6	*N-Sta*	Number of consecutive images, including the current one, along which the object remains static. If its value is 0, this means that the object is moving.

**Table 3:** Rate of usefulness of the tools used in Tests 2–5.

	**Test 1**	**Test 2**	**Test 3**	**Test 4**	**Test 5**
Mean	3.25	4.2	3.75	4.25	4.85
Variance	0.99	0.26	0.29	0.55	0.13
